# In the midst of a dangerous intersection with unclear therapeutic strategies: a challenging case of severe aortic stenosis

**DOI:** 10.1186/s12872-020-01533-x

**Published:** 2020-06-01

**Authors:** Guglielmo Gallone, Federico Landra, Fabrizio D’Ascenzo, Federico Conrotto, Roberta Casoni, Francesco Bruno, Pierluigi Omedè, Gianluca Alunni, Alessandro Andreis, Alessandro Vairo, Mauro Giorgi, Antonella Fava, Gaetano Maria De Ferrari

**Affiliations:** 1 Department of Medical Sciences, University of Turin, Città della Salute e della Scienza Hospital, Turin, Italy; 2 Division of Cardiology, Città della Salute e della Scienza, Corso Bramante 88/90, 10126 Turin, Italy

**Keywords:** Aortic stenosis, Cardiac amyloidosis, Transcatheter aortic valve intervention, Strain, Bone tracer scintigraphy, Heart team, Case report

## Abstract

**Background:**

One out of seven patients with severe aortic stenosis (AS) undergoing transcatheter aortic valve replacement (TAVR) may be affected by transthyretin cardiac amyloidosis (ATTR-CA), mostly presenting with low-flow low-gradient AS with mildly reduced ejection fraction. The complex interaction of these two pathologies poses specific diagnostic and management challenges. The prognostic implications of this clinical intersection are not defined yet. Moreover, whether TAVR may have a prognostic benefit in ATTR-CA patients with symptomatic severe AS remains unclear, posing doubts on the best management strategy in this increasingly recognized subset of patients.

**Clinical case:**

We present a case of an 87-year old man with low-flow low-gradient severe AS, for whom a diagnosis of ATTR-CA was suspected based on clinical and echocardiographic criteria specific to coexisting AS and ATTR-CA. The diagnosis was eventually confirmed by positive bone tracer scintigraphy imaging. Following in-depth Heart team discussion, integrating frailty and prognostic information from combined cardiomyopathy states, a decision was made to manage the patient’s severe AS conservatively.

**Conclusion:**

In the presented case, we deemed the natural history of ATTR-CA amyloidosis to negatively affect both the patient’ prognosis and procedural risk, adversing TAVR indication despite symptomatic severe AS. No clear evidence is currently available to guide decision making in this setting, advocating for prospective studies to clarify if TAVR may have a prognostic benefit in ATTR-CA - and which ATTR-CA - patients.

## Background

One out of seven patients with severe aortic stenosis (AS) undergoing transcatheter aortic valve replacement (TAVR) may be affected by transthyretin cardiac amyloidosis (ATTR-CA), mostly presenting with low-flow low-gradient AS with mildly reduced ejection fraction [1]. The complex interaction of these two pathologies poses specific diagnostic and management challenges. The prognostic implications of this clinical intersection are not defined yet. Moreover, whether TAVR may have a prognostic benefit in ATTR-CA patients with symptomatic severe AS remains unclear, posing doubts on the best management strategy in this increasingly recognized subset of patients.

## Case presentation

**Fig. 1:**
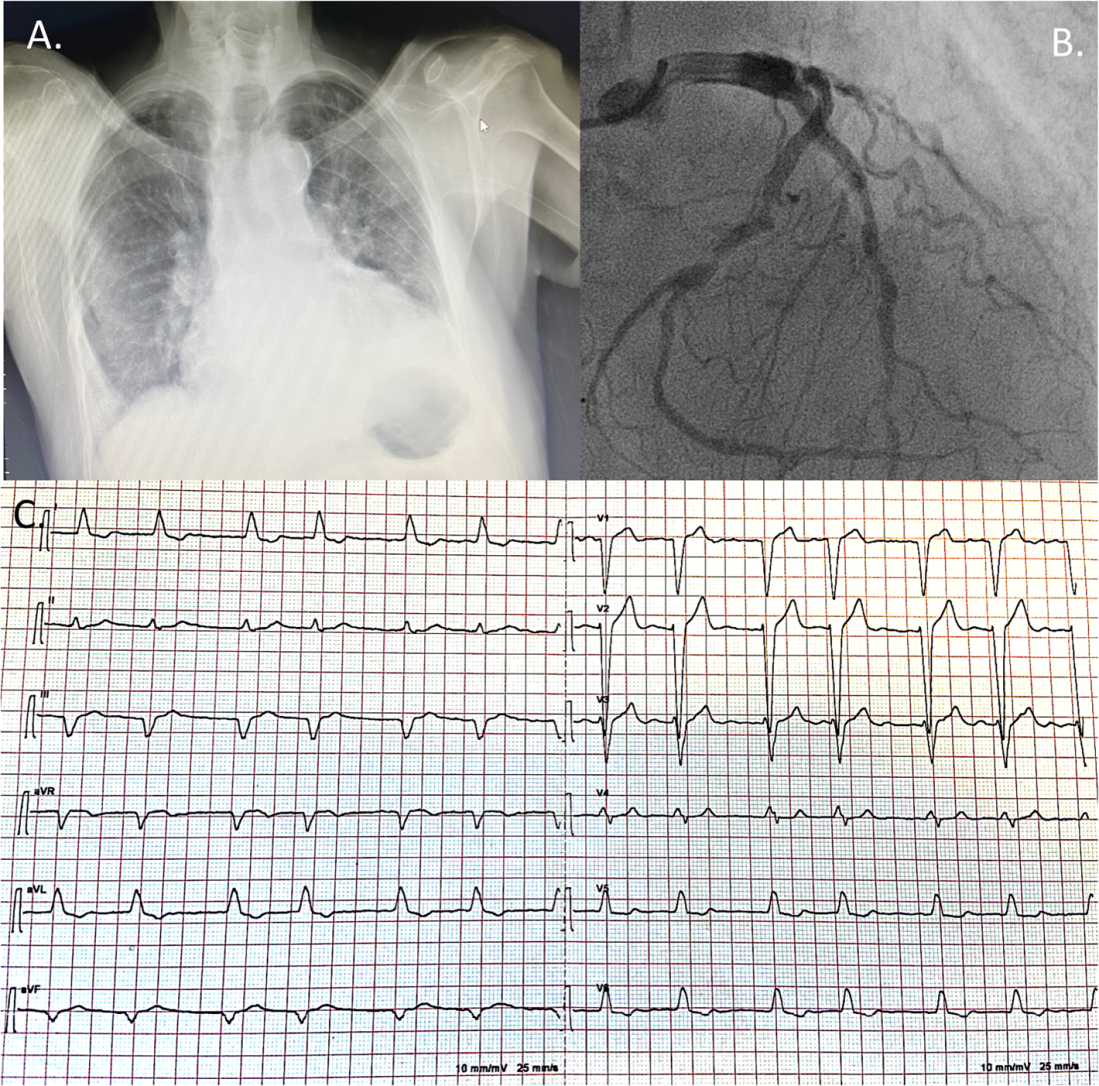
**a** Chest x-ray at patient hospital admission showing lung congestion and enlarged heart shadow (top left). **b** Coronary angiography showing diffuse disease with critical disease of the mid and distal left anterior descending coronary artery and of the proximal and distal circumflex coronary artery (top right). **c** Electrocardiogram showing atrial fibrillation and left bundle branch block (bottom)

A 87-year old man with a presenting diagnosis of hypertensive cardiomyopathy, orthostatic hypotension, moderate aortic stenosis, permanent atrial fibrillation, left bundle branch block and preserved ejection fraction (EF) (home therapy: valsartan 80 mg, bisoprolol 2.5 mg) was admitted to our Clinic with Canadian Cardiovascular Society III angina, New York Heart Association (NYHA) III functional class and signs of pulmonary and systemic venous congestion (Fig. 1).

**Fig. 2:**
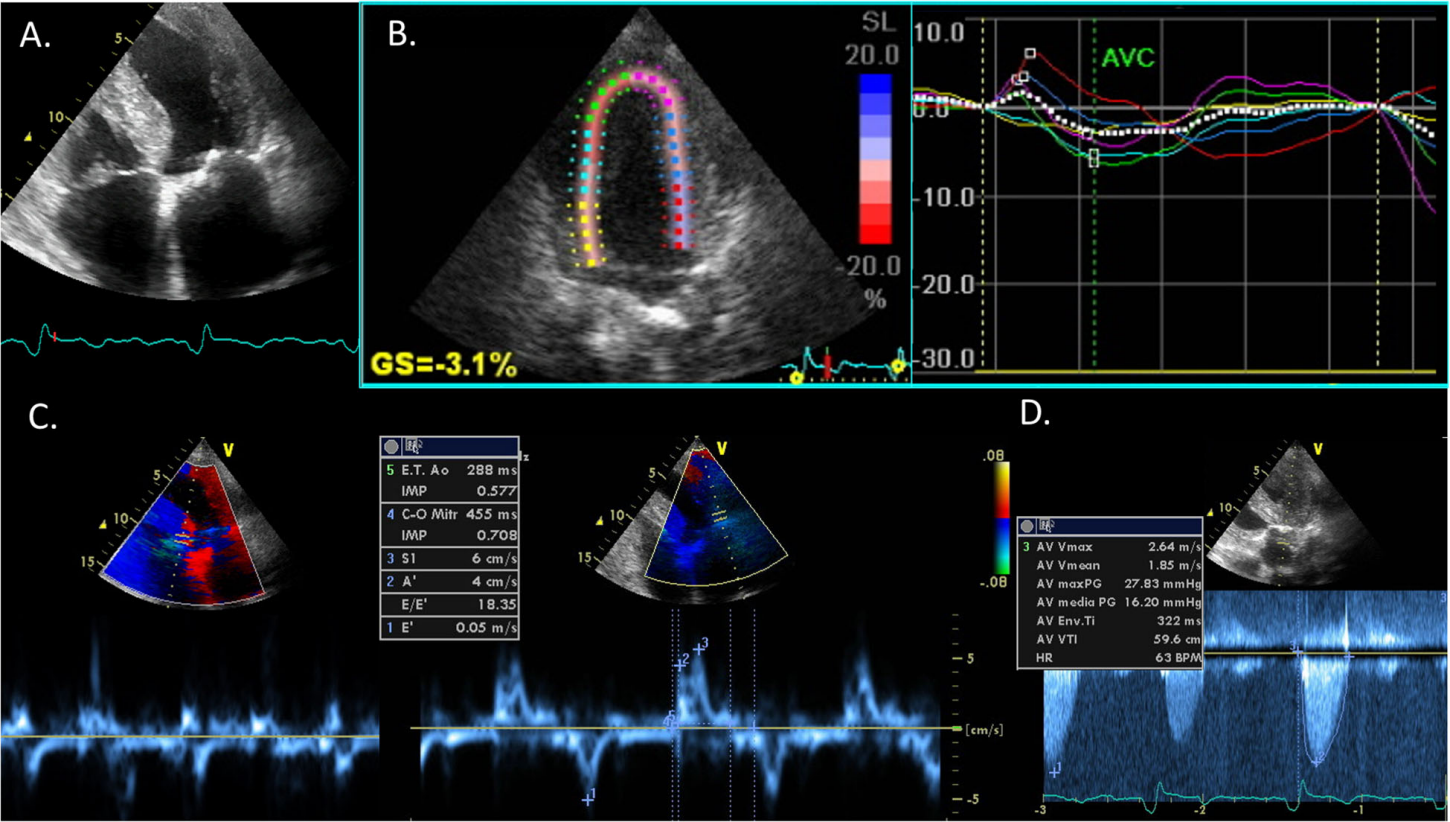
**a** Transthoracic echocardiographic apical 4-chamber view showing severe LV hypertrophy with myocardial granular sparkling, biatrial dilatation and atrial septal thickening. **b** Longitudinal strain analysis revealing severely impaired longitudinal systolic function without apical sparing. **c** Lateral and septal mitral annular tissue Doppler showing a severely reduced average S’. **d** Continuous wave doppler across LV outflow tract showing low gradient AS

Cardiac ultrasound documented left ventricle (LV) hypertrophy (septum 16 mm, posterior wall 15 mm, LV mass index 189 g/m^2^) with severe concentric remodeling (relative wall thickness 0.6), mildly reduced EF (50%), severely depressed global longitudinal strain (− 3.1%) with no relative apical sparing pattern and an average mitral annular Tissue Doppler S′ of 5.5 cm/sec. Second degree diastolic disfunction and severe biatrial dilatation were also apparent. A low QRS voltage-to-LV mass ratio by Sokolow-Lyon criteria was observed. Aortic valve evaluation revealed low flow-low gradient severe aortic stenosis (AS) (Vmax 2.6 m/sec, max/mean gradient 28/16 mmHg, indexed aortic valve functional area 0.4 cm^2^/m^2^, stroke volume index 21 ml/m^2^)((Fig. 2**)**, confirmed by computed tomography scan (aortic valve calcium score 2500).

Following successful decongestion and clinical improvement with intravenous diuretics and vasodilators, coronary angiography revealed critical multivessel diffuse disease, which was treated with complete percutaneous revascularization (two drug-eluting stents on the left anterior descending and circumflex coronary arteries).

**Fig. 3:**
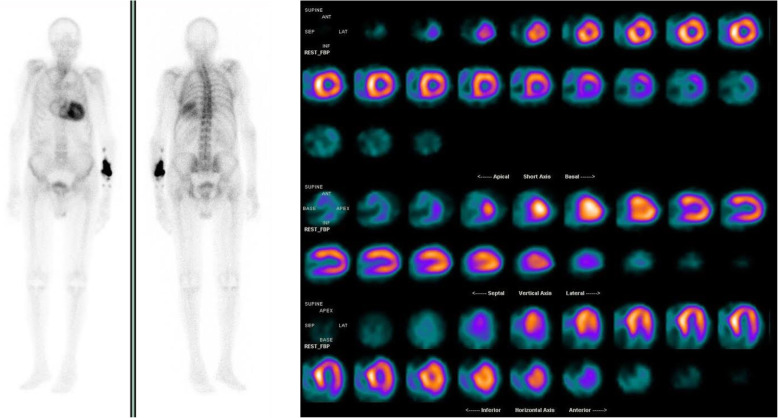
Bone tracer scintigraphy revealing high diffuse myocardial uptake (Perugini score 3)

Based on the low-flow low-gradient AS phenotype in the background of severe concentric hypertrophy, biatrial dilatation, S′ wave depression, conduction abnormalities, low QRS-voltage-to-LV-mass ratio and history of orthostatic hypotension, a diagnosis of amyloid cardiomyopathy was suspected. Bone tracer scintigraphy was performed revealing high diffuse myocardial uptake (Perugini score 3), diagnostic - in the context of an absent monoclonal protein on serum and urine exams (the patient had negative serum and urine immunofixation, and negative serum free light chain assay) - for transthyretin cardiac amyloidosis (ATTR-CA) [2] (Fig. 3).

The patient was unsuitable for ATTR-CA specific treatment because of current national reimbursement policies.

A Heart Team evaluation was carried. In consideration of the high procedural risk (EuroSCORE II 11.4%) transcatheter aortic valve replacement (TAVR) appeared indicated for severe AS treatment. However, considering the patient frailty (Essential Frailty Toolset score of 4/5: pre-procedural anemia (1 point), hypoalbuminemia (1 point), lower-extremity muscle weakness (2 points) [3]), the presence of ATTR-CA cardiomyopathy-specific markers of adverse prognosis (Mayo Clinic ATTR-CA stage III: high-sensitivity troponin T = 280 ng/L, NTproBNP = 6500 ng/L [4]; echocardiographic parameters heralding poor prognosis in ATTR-CA with severe AS: reduced stroke volume, severely reduced global longitudinal strain, increased filling pressures [5]), considering the clinical stability on oral diuretics following decongestion and revascularization and the patient preference not to proceed to further invasive management during the current hospitalization, the decision was made to discharge the patient on heart failure medical therapy, with an indication for TAVR re-evaluation in case of further acute decompensation. At last (6-month) follow-up the patient is alive, with clinical stability on medical therapy (NYHA IIb, no angina) and no ensuing cardiac rehospitalizations.

## Discussion and conclusions

ATTR-CA is a common under recognized condition among patients with severe AS, mostly presenting with low-flow low-gradient AS with mildly reduced EF [1]. A high index of suspicion is needed due to the overlapping clinical and echocardiographic presentations of AS patients with and without ATTR-CA. Suspicion should be based on a careful assessment of clinical features including older age, male sex, bilateral carpal tunnel syndrome, orthostatic hypotension, frequent hospitalizations for heart failure with preserved EF (prior to severe AS diagnosis) and conduction disorders. No association with bicuspid aortic valve disease is reported [6]. Classic echocardiographic signs suggestive of ATTR-CA are frequently inapparent or attributed to severe AS pathophysiology [7]. A relative longitudinal strain apical sparing pattern - while presenting high accuracy for ATTR-CA diagnosis in patients without severe AS - is usually absent in ATTR-CA patients with concomitant severe AS [1]. A tissue Doppler average mitral annular S′ of < 6 cm/s has been recently proposed as a 100% sensitive measure to screen TAVR candidates for ATTR-CA, with more than half patients with S′ < 6 cm/s having ATTR-CA as confirmed by bone tracer nuclear scintigraphy [1]. Only sparse retrospective evidence is available regarding prognosis of severe AS patients with ATTR-CA undergoing aortic valve intervention, mostly limited to surgical replacement and suggesting a high in-hospital and long-term mortality [7].

In this context, no solid evidence is currently available to inform the diagnostic strategies for ATTR-CA among patients with severe AS and, more importantly, whether and in which ATTR-CA patients TAVR (and which with TAVR device [8]) may have a prognostic benefit remains unclear.

In the presented case, a comprehensive heart team evaluation deemed the natural history of ATTR-CA to negatively affect both the patient’s prognosis and procedural risk, leading, along with consideration of patient preferences, to a lack of indication to TAVR during the index hospitalization despite severe AS. At mid-term follow-up, the patient was clinically stable without ensuing decompensation episodes, accordingly, clinical follow-up is ongoing. This management strategy, despite based on objective functional, laboratoristic and imaging parameters along with patient preferences, was based on a subjective weight of the numerous elements taken into considerations, as solid data regarding risk stratification and management of severe AS in patients with ATTR-CA are currently lacking. Indeed, no clear guidance to orientate decision making in this setting is available, advocating for prospective studies to clarify if TAVR may have a prognostic benefit in ATTR-CA - and which ATTR-CA-patients.

## Data Availability

Data sharing is not applicable to this article as no datasets were generated or analysed during the current study.

## Abbreviations

AS: Aortic stenosis
ATTR-CA: Transthyretin cardiac amyloidosis
EF: Ejection fraction
LV: Left ventricle
NYHA: New York heart association
TAVR: Transcatheter aortic valve replacement
